# Postoperative analgesia for pediatric craniotomy patients: a randomized controlled trial

**DOI:** 10.1186/s12871-019-0722-x

**Published:** 2019-04-11

**Authors:** Fei Xing, Li Xin An, Fu Shan Xue, Chun Mei Zhao, Ya Fan Bai

**Affiliations:** 1 Department of Anesthesia, Beijing Friendship Hospital, Capital Medical University, No.95 Yongan Road, Xicheng District, Beijing, 100050 China; 2 Department of Anesthesia, Beijing Tiantan Hospital, Capital Medical University, Beijing, China

**Keywords:** Pain, Postoperative, Child, Craniotomy

## Abstract

**Background:**

Pain is often observed in pediatric patients after craniotomy procedures, which could lead to some serious postoperative complications. However, the optimal formula for postoperative analgesia for pediatric neurosurgery has not been well established. This study aimed to explore the optimal options and formulas for postoperative analgesia in pediatric neurosurgery.

**Methods:**

Three hundred and twenty patients aged 1 to 12-years old who underwent craniotomy were randomly assigned to receive 4 different regimens of patient-controlled analgesia. The formulas used were as follows: Control group included normal saline 100 ml, with a background infusion of 2 ml/h, bolus 0.5 ml; Fentanyl group was used with a background infusion of 0.1–0.2 μg/k·h, bolus 0.1–0.2 μg/kg; Morphine group was used with a background infusion of 10–20 μg/kg·h, bolus 10–20 μg/kg; while Tramadol group was used with a background infusion of 100–400 μg/kg·h, bolus 100–200 μg/kg. Postoperative pain scores and analgesia-related complication were recorded respectively. Comparative analysis was performed between the four groups.

**Results:**

In comparison of all groups with each other, lower pain scores were shown at 1 h and 8 h after surgery in Morphine group versus Tramadol, Fentanyl and Control groups (*P* < 0.05). Both Tramadol and Fentanyl groups showed lower pain scores in comparison to Control group (*P* < 0.05). Nausea and vomiting were observed more in Tramadol group in comparison to all other groups during the 48 h of PCIA usage after operation (*P* = 0.020). Much more rescue medicines including ibuprofen and morphine were used in Control group (CI = 0.000–0.019). Changes in consciousness and respiratory depression were not observed in study groups. Moderate-to-severe pain was observed in a total of 56 (17.5%) of the study population. Multiple regression analysis for identifying risk factors for moderate-to-severe pain revealed that, younger children (OR = 1.161, 1.027–1.312, *P* = 0.017), occipital craniotomy (OR = 0.374, 0.155–0.905, *P* = 0.029), and morphine treatment (OR = 0.077, 0.021–0.281, *P* < 0.001) are the relevant factors.

**Conclusions:**

Compared with other analgesic projects, PCIA or NCIA analgesia with morphine appears to be the safest and most effective postoperative analgesia program for pediatric patients who underwent neurosurgical operations.

**Trial registration:**

Chinese Clinical Trial Registry. No: ChiCTR-IOC-15007676. Prospective registration. http://www.chictr.org.cn/index.aspx.

**Electronic supplementary material:**

The online version of this article (10.1186/s12871-019-0722-x) contains supplementary material, which is available to authorized users.

## Background

Pain after craniotomy is a frequent source of concern and controversy. Over the past decade, several studies—primarily in adult patients—have revealed that moderate-to-severe pain is common in patients after major craniotomy [1–4]. Furthermore, very few studies have assessed pain or analgesic requirements in pediatric patients following neurosurgery, primarily due to fear of opioid analgesics masking alterations in the postoperative neurological exam and delaying detection of intracranial postoperative complications [5–7]. Postoperative pain in pediatric neurosurgical patients appears to be underestimated often [6, 7]. Inadequate pain control in children after major craniotomy may contribute to significant anxiety, hypertension, shivering, and emesis, which may in turn increase intracranial pressure and cause bleeding [8, 9]. Therefore, although frequently overlooked, postoperative analgesia in children after craniotomy is important.

Opioids are the most frequently prescribed analgesics for moderate-severe pain. However, they may be associated with side effects such as nausea, vomiting, pruritus, respiratory depression, and neurological alterations [10–12]. In particular, treatment of postoperative pain after craniotomy without affecting neurological status remains a major clinical problem. Recent studies have reported neurosurgical postoperative pain in pediatric patients can be managed with opioids without neurologic deterioration [6, 7]. Nevertheless, these reports are mostly small cohort studies and reviews. So far, no prospective randomized controlled trial has been conducted on postoperative pain in pediatric neurosurgery.

Therefore, the aim of this prospective, randomized, controlled study is to assess the safety and efficacy of different postoperative pain treatment in pediatric craniotomy patients. We selected the most commonly used postoperative analgesic formulas in clinical practice in accordance with our previous research, and assumed that one of the postoperative pain treatment formulas has the best analgesic effect and no related side effect for 1–12 years old children undergoing craniotomy , so as to find an optimal formula for pediatric neurosurgery postoperative analgesia.

## Methods

### Study design and participants

This randomized controlled clinical trial was approved by the Institutional Review Board of Beijing Tiantan Hospital Affiliated to Capital Medical University (Beijing, China, KY2015–009-01). Written informed consent was obtained from all patients’ parents. This study was conducted at a single tertiary medical center (Beijing Tiantan Hospital) and indexed in the Chinese Clinical Trial Registry (http://www.chictr.org.cn/index.aspx, ChiCTR-IOC-15007676).

The inclusion criteria were as follows: Patients aged 1–12 years, with American Society of Anesthesiologists physical status grades I–III undergoing open craniotomy procedures. Eligible subjects included patients undergoing surgery for brain tumors, craniofacial reconstruction and vascular malformations. Exclusion criteria included: Mental disorders; unsuitability for extubation; and development of hematomas or severe brain edema 3 days after surgery, requiring a subsequent operation. Additionally, we excluded patients with a history of allergy to opioids or other anesthetics, and those with a history of substance abuse. Patients were enrolled in this study only after obtaining written informed consent from their parents.

### Anesthesia

Standard monitoring was implemented in the operating room. All children were monitored for non-invasive blood pressure (BP), heart rate (HR) and pulse oximetry (SpO_2_); as well as invasive arterial pressure (ARP), end-tidal carbon dioxide partial pressure (P_ET_CO_2_), and minimum alveolar concentration (MAC). Midazolam 0.025–0.075 mg/kg and methylprednisolone sodium succinate 1–2 mg/kg were given before surgery. If necessary, patients were given oral midazolam 0.5 mg/kg to reduce anxiety before venous access.

Anesthesia was induced with the following approximate doses: Propofol (2 mg/kg), cisatracurium (0.2 mg/kg), and sufentanyl (0.3 μg/kg) or fentanyl (3 μg/kg). In patients aged < 5 years or those unable to cooperate with the anesthesiologist, tracheal intubation was performed under induction with 6–8% sevoflurane inhalation before peripheral venous access. Prior to surgical incision, local infiltration with 0.5% ropivacaine was performed at the surgical site, and surgical pin sites was placed. Anesthesia was maintained with 0.5 MAC sevoflurane at an inhalational concentration of 2–3%, and an intravenous infusion with remifentanil 0.1–0.2 μg/kg/min and propofol 3–5 mg/kg/h. Mean arterial blood pressure and heart rate were maintained within 20% of baseline measures. 30 min before the end of the operation, additional sufentanyl 5 μg or fentanyl 0.5–1 μg/kg was administered, while inhalation of sevoflurane and the infusion of remifentanil and propofol was stopped at the end of the operation. The parameters for mechanical ventilation were set to volume control with a tidal volume of 8–10 ml/kg and a respiratory rate of 14–20 times/min. Controlled mechanical ventilation maintained P_ET_CO_2_ of 30–35 mmHg using a 50% oxygen-air gas mixture. Additional rocuronium was administered, if needed, to maintain a train-of-four count of 2–3 intraoperatively. Whether patients received a central and arterial cannula after anesthesia induction was according to the needs of the operation.

### Postoperative pain treatment protocol

After surgery, patients aged 1–6 years received a pump for nurse-controlled intravenous analgesia (NCIA), while those aged 7–12 received one for patient-controlled intravenous analgesia (PCIA). Based on our previous cohort study of pediatric postoperative analgesia, we found that only 12% in 1–6 years old and 58% in 7–12 years old patients used PCIA or NCIA after craniotomy [13]. Same results were obtained in another cohort study performed by Maxwell LG^7^. That means that single intravenous administration after operation is a common method of postoperative analgesia in pediatric neurosurgery. So in our study we used saline in PCIA/NCIA plus rescue medicine as our control group.

The regimens of PCIA or NCIA used the following formulas: Control (group C) included normal saline 100 ml, with a continuous background infusion of 2 ml/h, bolus 0.5 ml; Fentanyl (group F) was used with a loading dose of 0.5 μg/kg, a single bolus dose of 0.1–0.2 μg/kg, and a background dose 0.1–0.2 μg/k·h; Morphine (group M) was used with a loading dose of 50 μg/kg, a single bolus dose of 10–20 μg/kg, and a background dose of 10–20 μg/kg·h; while Tramadol (group T) was used with a loading dose of 500 μg/kg, a single bolus dose of 100–200 μg/kg, and a background dose of 100–400 μg/kg·h. The bolus locking time was 15 min. The total volume contained in the analgesia pump was adjusted to 100 ml with normal saline and 0.4 mg/kg of ondansetron. Patients would receive additional doses of ondansetron if they reported nausea or experienced vomiting. The type and dosed of medicines used in pump were converted to their respective milligram morphine equivalents (MME) using standardized conversion factors (1 mg of Fentanyl = 100 MME, 1 mg of tramadol = 0.1 MME) [14].

As a rescue medicine, ibuprofen suspension (20 mg/ml ibuprofen) was oral administered in the postoperative period in doses of 0.3 ml/kg for moderate pain (defined as a pain score ≥ 4 and < 7) within 48 postoperative hours. If the POPI is severe pain (defined as a pain score ≥ 7) or the first administration of ibuprofen couldn’t comfort the patient within 30 min, another rescue medicine intravenous morphine 0.02 mg/kg would be administered through peri vein. All rescue medicines were recorded.

### Evaluation of pain intensity

The primary outcome of this study was postoperative pain intensity (POPI). According to the particular characteristics of each patient, we adopted different evaluation methods for POPI. Patients aged 1–6 years were evaluated by the Faces, Legs, Activity, Cry and Consolability Scale (FLACC, 0–10 scores) and the Wong-Baker Faces Scale (WBFS). For patients aged 7–12 years, both the numeric rating scale (NRS) and the Wong-Baker Faces Scale (WBFS) were used. The FLACC is a behavioral pain assessment tool that was developed to provide a simple and consistent evaluation method for these cases [15], while the WBFS is a self-reported pain assessment tool, currently considered the preferred alternative for pain assessment in children [16]. The WBFS is comprised of a series of facial images, in which the face that depicts the most pain indicates the “worst pain imaginable” and the happiest face indicates “no pain” [15]. The numeric rating scale (NRS) is a self-reported measure of pain intensity comprised of a line marked with numbers 0–10, in which 0 is “no pain” and 10 is the “worst pain imaginable” [17]. POPI ratings were measured at 1, 2, 4, 16, 24, 36 and 48 h after surgery by the same observer. Moderate POPI was defined as a median pain score ≥ 4 and < 7 on the WBFS, FLACC or NRS scales. Severe pain was defined as a median pain score ≥ 7.

### Randomization and blinding

Participants were randomly assigned 1:1:1:1 among four groups. The randomization schedule was generated by an independent investigator through a computerized random-number sequence. A specially selected nurse was informed of the group assignments and prepared the postoperative analgesia pumps according to the patients’ weights. Anesthesiologists were blinded to grouping information. Physicians responsible for postoperative follow-up were also blinded to the grouping.

### Data collection

Demographic data were recorded, including age, sex, height, weight, disease information, primary diagnosis, patients' medical history, and medications. Perioperative and anesthetic management information were also collected including: operation type; preoperative anesthetic medications; induction medications; intra-operative anesthetic medications; duration of surgery and anesthesia; and number of rescue medicine administrations.

The primary outcomes included pain scores at 1, 2, 4, 16, 24, 36 and 48 h after surgery. Secondary outcomes included the incidence of changes in consciousness, nausea, vomiting, pruritus, respiratory depression, and addition of perioperative acetaminophen. Nausea and vomiting were recorded if episodes of patient emesis were reported on nursing flow sheets, or if anti-emetic therapy was required. Respiratory depression was operationalized as a clinically significant decline in respiratory rate which required intervention, with SpO_2_ < 92%.

### Sample size and statistical analysis

Continuous variables were described as median and interquartile range (IQR) or mean and standard deviation (SD), as appropriate. Categorical variables (sex, site of craniotomy) were presented as frequencies and percentages. The chi-square test was used for comparing proportions, and one-way analysis of variance (ANOVA) was used for comparing continuous variables between groups. Because the POPI of patients was ranked data, we used Kruskal-Wallis H-test to compare the differences of POPI among all groups. If *P* < 0.05, Dunnett’s T3 test was used to compare the differences of POPI between any two groups.

Our previous cohort study on POPI in pediatric craniotomy patients found that the incidence of moderate POPI in children to be approximately 45%. A final sample size was calculated based on the hypothesis that PCIA could reduce the incidence of moderate POPI at least 30%. A sample size of 36 patients was calculated to have a significance of 5% and a power of 80%, increased to 40 after considering a 10% maximal dropout rate.

Step-wise multivariate logistic regression was used to identify predictors for moderate POPI, with results presented as odds ratios (OR) and 95% confidence intervals (CI). Statistical analysis was performed using SPSS (version 22, BEIJING, Capital Medical University). All statistical tests were two-sided, and results were considered statistically significant when *P* < 0.05.

## Results

### Baseline characteristics

**Fig. 1:**
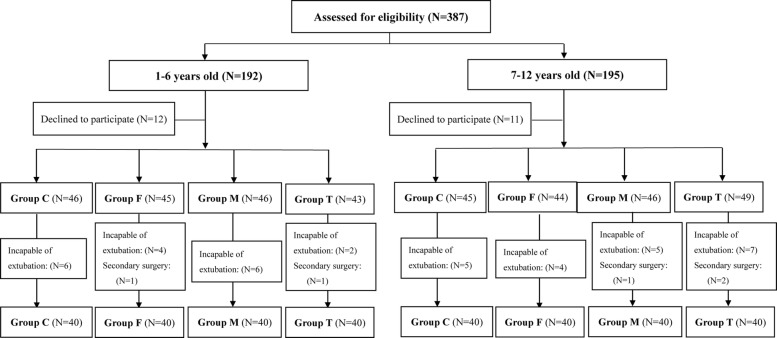
Consort flow chart of participants

A total of 387 consecutive patients who underwent major craniotomy were screened for study participation between January 2016 and June 2018; 192 of which were in a younger group (aged 1–6 years) and 195 in an older group (aged 7–12 years). In the younger group, 12 cases refused informed consent, 18 children remained intubated for surgical reasons, and 2 children required a second operation due to postoperative hematoma. Therefore, 160 patients were ultimately included. In the older group, 11 cases refused informed consent, 21 children remained intubated for surgical reasons, and 3 children required a second operation within 48 h of surgery. Therefore, 160 patients (91 males and 69 females) were finally included. An explanatory flow chart is depicted in Fig. 1.

The baseline clinical characteristics in all pediatric patients are presented in Tables 1. No significant differences were found regarding these variables among the four PCIA regimens either in the younger or older pediatric patients.

**Table 1:** Baseline Data For all Pediatric Patients (1–12 years old, *X ± SD*)

	1–6 Years Old Patients				7–12 Years Old Patients			
	Group C	Group F	Group M	Group T	Group C	Group F	Group M	Group T
Age (yr)	4.00 ± 1.77	3.50 ± 1.54	4.05 ± 1.45	3.70 ± 1.64	8.97 ± 2.92	9.21 ± 1.68	8.51 ± 2.69	9.29 ± 1.60
Sex (male/female)	24/16	27/13	24/16	22/18	25/15	18/22	24/16	24/16
Height (m)	1.07 ± 0.16	1.03 ± 0.13	1.05 ± 0.11	1.06 ± 0.14	1.42 ± 0.15	1.33 ± 0.13	1.38 ± 0.14	1.40 ± 0.13
Weight (kg)	19.8 ± 5.4	17.4 ± 4.7	18.9 ± 6.0	18.3 ± 6.3	37.7 ± 15.5	32.6 ± 8.5	34.6 ± 10.9	36.12 ± 11.5
Craniotomy site(*n* / %)								
Forehead	24 (60)	19 (47.5)	18 (45)	17 (42.5)	22 (55.0)	23 (57.5)	21 (52.5)	19 (47.5)
Frontotemporal	2 (5)	6 (15)	9 (22.5)	3 (7.5)	4 (10.0)	3 (7.5)	7 (17.5)	5 (12.5)
Frontoparietal	1 (2.5)	1 (2.5)	2 (5)	2 (5)	3 (7.5)	2 (5.0)	2 (5.0)	3 (7.5)
Temporal occipital	1 (2.5)	0 (0)	1 (2.5)	2 (5)	1 (2.5)	0 (0.0)	1 (2.5)	1 (2.5)
Occipital	11 (27.5)	11 (27.5)	9 (22.5)	15 (37.5)	10 (25)	11 (27.5)	8 (20.0)	11 (27.5)
Temporal-parietal occipital	1 (2.5)	3 (7.5)	1 (2.5)	1 (2.5)	0 (0.0)	1 (2.5)	1 (2.5)	1 (2.5)
VP shunt surgery (Y/N)	5/35	8/32	7/33	7/33	7/33	4/36	1/39	7/33
Durations of surgery (min)	217 ± 81	229 ± 112	212 ± 67	215 ± 63	299 ± 59	205 ± 134	219 ± 49	229 ± 60
Durations of anesthesia (min)	320 ± 98	403 ± 73	310 ± 80	308 ± 74	331 ± 77	290 ± 81	311 ± 63	328 ± 66
Bleeding (ml)	161 ± 199	109 ± 72	136 ± 168	118 ± 93	133 ± 80	113 ± 95	163 ± 141	129 ± 80
Anesthesia maintenance phase								
Propofol (mg)	225 (155, 337)	190 (140, 367)	230 (160, 300)	220 (152, 340)	525 (292, 672)	350 (215, 555)	340 (200, 490)	280 (160, 480)
Remifentanil (mg)	0.63 ± 0.34	0.61 ± 0.46	0.49 ± 0.25	0.68 ± 0.36	1.07 ± 0.59	0.82 ± 0.45	0.91 ± 0.57	1.24 ± 1.44
Sevoflurane (ml)	30 ± 20	28 ± 22	28 ± 13	24 ± 13	29 ± 12	22 ± 12	26 ± 11	27 ± 11

No significant difference of baseline characteristics was observed

### Postoperative pain intensity

Pain intensity was evaluated at 1, 2, 4, 16, 24, 36 and 48 h after surgery (Table 2). In the younger patients, over time, the pain intensity gradually decreased, and increased slightly at 24 h after surgery (Additional file 1: Figure S1 and S2). The differences of WBFS/FLACC scores were significantly among all groups at 1–8 h (*P* < 0.05) by Kruskal-Wallis test. Through Dunnett’s T3 test, lower pain scores (Both WBFS/FLACC Scores) were shown at 1 h and 8 h after surgery in Morphine Group versus Tramadol, Fentanyl and Control groups (*P* < 0.05). Both Fentanyl and Tramadol groups showed lower pain scores in comparison to Control group (*P* < 0.05), and there is no significant difference in pain scores between the Fentanyl and Tramadol groups (*P* > 0.05) (Table 2, Additional file 1: Table S1).

**Table 2:** Postoperative Pain Scores For 1–6 Years Old Younger Pediatric Patients (median (interquartile range))

	Group C		Group F		Group M		Group T		95% CI	
	FLACC	WBFS	FLACC	WBFS	FLACC	WBFS	FLACC	WBFS	*CI 1*	*CI 2*
1 h	3 (2, 5)	4 (2, 6)	2 (1, 3.5) *	2 (2, 4) *	2 (1.25, 2) *^∆^	2 (2, 2) *^∆^	2 (2, 4) *	2 (2, 4) *	0.000–0.019	0.000–0.019
2 h	3 (2, 5)	4 (2, 6)	2 (0, 2) *	2 (1.5, 2) *	2 (1, 2) *^∆^	2 (2, 2) *^∆^	2 (2, 3) *	3 (2, 4) *	0.000–0.019	0.000–0.019
4 h	2 (0.5, 4)	2 (2, 4)	1.5 (0, 2) *	2 (0, 2) *	1 (0, 2) *^∆^	0 (0, 2) *^∆^	2 (0.25, 2) *	2 (0.5, 2) *	0.000–0.019	0.000–0.018
8 h	0 (0, 2.5)	0 (0, 3)	0 (0, 1.5) *	0 (0, 2) *	0 (0, 0.5) *^∆^	0 (0, 0) *^∆^	2 (0, 2) *	2 (0, 2) *	0.001–0.049	0.000–0.018
16 h	1 (0, 3.5)	2 (0, 4)	0 (0, 2)	0 (0, 2)	0 (0, 1)	0 (0, 2)	0.5 (0, 2)	1 (0, 2)	0.012–0.075	0.030–0.108
24 h	2 (2, 3)	2 (2, 4)	1.5 (0, 2)	2 (0, 2)	2 (0, 2)	2 (0, 3.5)	2 (0, 2)	2 (0, 2)	0.000–0.018	0.030–0.108
36 h	0 (0, 0.5)	0 (0, 1)	0 (0, 0)	0 (0, 0)	0 (0, 0)	0 (0, 0)	0 (0, 2)	0 (0, 2)	0.054–0.146	0.000–0.019
48 h	1.5 (0, 2)	2 (0, 2)	0 (0, 0.75)	0 (0, 2)	0 (0, 1)	0 (0, 2)	0 (0, 2)	0 (0, 2)	0.012–0.075	0.008–0.067

*CI 1*: 95% Confidence interval for FLACC score among four groups by Kruskal-Wallis H-test;

*CI 2*: 95% Confidence interval for WBFS score among four groups by Kruskal-Wallis H-test

*P < 0.05, the difference was significant compared with group C by Dunnett’s T3 test;

∆ *P* < 0.05, compared with group F and M, the FLACC and WBFS in group M was significant lower through Dunnett’s T3 test

In the 7–12 years older patients, a similar trend was observed (Additional file 1: Figure S3 and S4), with WBFS/NRS scores being significantly lower at 1–16 h in Morphine group (Table 3, *P* < 0.05). There was no significant difference between Fentanyl and Tramadol groups (Additional file 1: Table S2, *P* > 0.05), but they were lower than Control group (*P* < 0.05).

**Table 3:** Postoperative Pain Scores For 7–12 Years Old Senior Pediatric Patients (median (interquartile range))

	Group C		Group F		Group M		Group T		95% CI	
	WBFS	NRS	WBFS	NRS	WBFS	NRS	WBFS	NRS	*CI 1*	*CI 2*
1 h	4 (2, 6)	4 (2, 5)	2 (2, 4) *	2 (2, 3.3) *	2 (2, 4) *^∆^	2 (2, 3) *^∆^	2 (2, 4) *	2 (2, 4) *	0.000–0.021	0.000–0.044
2 h	4 (2, 4)	3 (2, 4)	2 (2, 4) *	2 (2, 4) *	2 (2, 2) *^∆^	2 (2, 3) *^∆^	4 (2, 4) *	4 (2, 4) *	0.038–0.129	0.018–0.094
4 h	2 (0, 4)	2 (0, 4)	2 (0, 2.5) *	2 (0, 3) *	0 (0, 2) *^∆^	0 (0, 2) *^∆^	2 (2, 4) *	2 (2, 4) *	0.000–0.021	0.000–0.021
8 h	2 (0, 2)	2 (0, 2)	0 (0, 2) *	0 (0, 2) *	0 (0, 0) *^∆^	0 (0, 0) *^∆^	2 (0, 2) *	2 (0, 2) *	0.000–0.021	0.000–0.021
16 h	2 (0, 2)	2 (0, 2)	0 (0, 2) *	0 (0, 2) *	0 (0, 2) *^∆^	0 (0, 2) *^∆^	2 (0, 2) *	2 (0, 2) *	0.001–0.055	0.000–0.021
24 h	2 (2, 4)	2 (2, 4)	2 (2, 2)	2 (1, 2)	2 (0, 2)	2 (0, 2)	2 (1, 2)	2 (1, 2)	0.500–0.661	0.507–0.668
36 h	0 (0, 0)	0 (0, 1)	0 (0, 0)	0 (0, 0)	0 (0, 0)	0 (0, 0)	0 (0, 2)	0 (0, 1)	0.485–0.648	0.346–0.508
48 h	2 (0, 2)	2 (0, 2)	0 (0, 2)	0 (0, 2)	0 (0, 2)	0 (0, 2)	0 (0, 2)	0 (0, 2)	0.044–0.138	0.023–0.103

*CI 1*: 95% Confidence interval for WBFS score among four groups by Kruskal-Wallis H-test;

*CI 2*: 95% Confidence interval for NRS score among four groups by Kruskal-Wallis H-test

*P < 0.05, the difference was significant compared with group C by Dunnett’s T3 test;

∆ *P* < 0.05, compared with group F and M, the WBFS and NRS in group M was significant lower through Dunnett’s T3 test

### Total amount of medicines used in PCIA or NCIA or for remedy

Total amount of medicines used in the postoperative analgesia pump was calculated. After all kinds of medicines converted to their respective milligram morphine equivalents (MME) using standardized conversion factors, the average morphine equivalent amount in each day was similar between Fentanyl and Morphine groups, and in Tramadol group was a little bit higher (Table 4). As rescue medicines, the total amount and cases of ibuprofen and morphine used in Control group were much higher than that in Fentanyl, Morphine and Tramadol groups, this result was similar in both 1–6 years old patients and 7–12 years old patients.

**Table 4:** Total amount of medicines used in PCIA or NCIA or for remedy (1–12 years old, *X ± SD*)

	1–6 Years Old Patients				7–12 Years Old Patients			
	Group C	Group F	Group M	Group T	Group C	Group F	Group M	Group T
Average total medicines use in PCIA or NCIA pump (mcg/kg/d)								
1st day	0	5.23 ± 1.21	472 ± 85	7210 ± 1560	0	5.65 ± 1.54	504 ± 105	9010 ± 2060
2nd day	0	5.05 ± 1.04	495 ± 92	6780 ± 1050	0	5.54 ± 0.98	493 ± 118	8280 ± 1150
Morphine equivalents	0	514 ± 112	486 ± 90	699 ± 164	0	560 ± 112	502 ± 113	864 ± 154
Total amount of Rescue medicines used in each group (48 h)								
Ibuprofen (P.O., mg)	3120	920*	540*	1290*	6000	400*	840*	650*
Ibuprofen (cases **/** %)	26/65%	9/22.5%*	5/12.5%*	12/30%*	27/67.5%	2/5%*	4/10%*	3/7.5%*
Comparison of Ibuprofen	**X2 = 27.473**		**95%CI = 0.000–0.019**		**X2 = 54.504**		**95%CI = 0.000–0.019**	
Morphine (I.V., mg)	4.8	0.4*	0*	1.1*	11.1	0*	0*	2.2*
Morphine (cases / %)	12/30%	1/2.5%*	0*	3/7.5%*	15/37.5%	0*	0*	4/10%*
Comparison of Morphine	**X2 = 20.879**		**95%CI = 0.000–0.019**		**X2 = 31.848**		**95%CI = 0.000–0.019**	

**Average total medicines use in PCIA or NCIA pump (mcg/kg/d**):

**1st day (2nd day),** Group F = Total Fentanyl per kg used in pump during the first postoperative day (second day); Group M = Total Morphine per kg used in pump during the first postoperative day (second day); Group T = Total Tramadol per kg used in pump during the first postoperative day (second day)

**Morphine equivalents:** All medicines converted to their morphine equivalents, and the average total morphine per kg used in pump per day

**Total amount of Rescue medicines used in each group (48 h):** As rescue medicines, the total amount and cases(%) of ibuprofen or morphine used in one group; *P < 0.001 compared with Group C

### Identical factors associated with moderate postoperative pain intensity

**Fig. 2:**
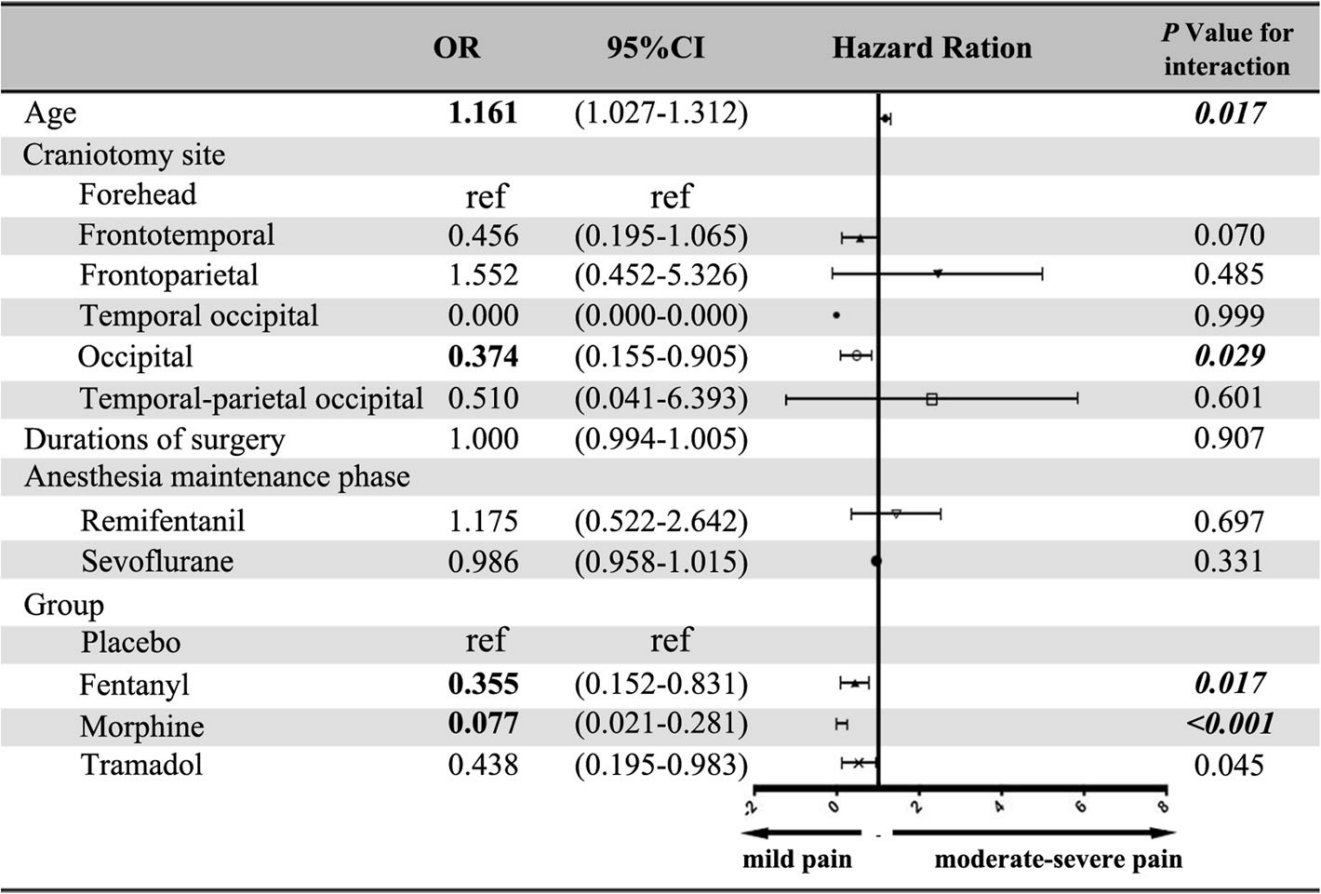
OR (95% CI) for the associations between factors and moderate-severe POPI (≥4). A multiple factor regression analysis was conducted including all factors with *P* < 0.2 in univariate logistic regression analysis results. Craniotomy site expressed different craniotomy approaches. Durations of surgery meant the length of operation. Remifentanil means the total amount of the use of remifentanil during anesthesia. The total amount of sevoflurane use is calculated based on the patients’ inhaled concentration and fresh gas flow and time. A total of 14 factors were included. For multiple groups of categorical variables, we chose one of them as the reference. So, we chose forehead in craniotomy site and placebo group in groups as reference. Age, occipital craniotomy, give fentanyl PCIA or NCIA, or give morphine PCIA or NCIA, were correlated risk factors of moderate-severe pain

Moderate-to-severe pain was observed in a total of 56 (17.5%) of the all study population. There were 26 patients in 1–6 years old groups (26/160, 16.25%) and 30 patients in 7–12 years old groups (30/160, 18.75%). Only 3 children experienced severe pain in Control group and 1 child in Tramadol group among 1–6 years old patients. And there were 5 patients in Control Group experienced severe pain among 7–12 years old patients. Single regression analysis for identifying risk factors revealed that, older age, site of craniotomy, dose of remifentanil and PCIA group were associated with moderate-to-severe POPI (Table 5). Then, multiple factor regression analysis was conducted on factors with *P* > 0.2. Multiple regression analysis for identifying risk factors for moderate-to-severe pain revealed that, younger children (OR = 1.161, 1.027–1.312, *P* = 0.017), occipital craniotomy (OR = 0.374, 0.155–0.905, *P* = 0.029), and fentanyl treatment (OR = 0.355, 0.152–0.831, P = 0.017), or morphine treatment (OR = 0.077, 0.021–0.281, *P* < 0.001) are the relevant factors (Fig. 2).

**Table 5:** Univariate Logistic Regression Analysis Of Influencing Factors Of Pain Scores For 1–12 Years Old Patients

	WBFS< 4 (*n* = 264)	WBFS≥4 (*n* = 56)	OR	95%CI	*P*
Age (yr)	6.25 ± 3.16	7.08 ± 3.26	1.085	(0.988–1.192)	0.088
Sex (male/female)	151/113	37/19	1.455	(0.782–2.708)	0.237
Height (m)	1.20 ± 0.21	1.25 ± 0.23	0.981	(0.875–1.100)	0.741
Weight (kg)	26.1 ± 12.5	28.1 ± 11.7	1.013	(0.990–1.036)	0.275
Craniotomy site(*n* / %)					
Forehead	75 (28.4)	25 (45.3)	Ref	Ref	
Frontotemporal	93 (35.2)	13 (22.6)*	0.418	(0.200–0.877)	***0.021***
Frontoparietal	13 (4.8)	6 (11.3)	1.400	(0.475–4.127)	0.542
Temporal occipital	5 (2.0)	0 (0)	0.000	(0.000–0.000)	0.999
Occipital	74 (28.0)	11 (18.9)*	0.400	(0.179–0.894)	***0.026***
Temporal-parietal occipital	4 (1.6)	1 (1.9)	0.700	(0.075–6.565)	0.755
0.5% Ropivacaine for local anesthesia (Y/N)	14/250	3/53	0.914	(0.251–3.327)	0.892
VP shunt surgery (Y/N)	42/222	8/48	1.071	(0.470–2.444)	0.870
Durations of surgery (min)	222 ± 87	205 ± 62	0.997	(0.992–1.001)	0.174
Durations of anesthesia (min)	315 ± 80	300 ± 68	0.997	(0.993–1.001)	0.201
Bleeding (ml)	132 ± 117	134 ± 158	1.000	(0.998–1.002)	0.913
Anesthesia maintenance phase					
Propofol (mg)	315 ± 224	326 ± 242	1.000	(0.999–1.001)	0.747
Remifentanil (mg)	0.73 ± 0.47	0.84 ± 0.57*	1.644	(1.016–2.658)	***0.043***
Sevoflurane (ml)	23.3 ± 17.1	19.3 ± 14.9	0.983	(0.964–1.004)	0.106
Group (n / %)					
Placebo	55 (20.8)	24 (43.4)	Ref	Ref	
Fentanyl	71 (26.8)	12 (20.8)*	0.364	(0.167–0.793)	***0.011***
Morphine	75 (28.4)	4 (7.5)*	0.083	(0.024–0.291)	***< 0.001***
Tramadol	63 (24.0)	16 (28.3)	0.500	(0.238–1.050)	***0.067***

### Analgesia-related complications

There was no significant difference in complications during recovery among the four groups, either in the younger or older children. In Tramadol group, 11 children suffered nausea (27.5%) and 19 children suffered vomiting (47.5%) within 48 h after surgery, which were significantly higher than that in Fentanyl, Morphine and Control groups (Additional file 1: Table S3, *P* < 0.05). Changes in consciousness and respiratory depression were not observed in study groups (*P* = 0.061). In the younger patients, much children needed ibuprofen suspension and intravenous morphine as rescue medicine in the Tramadol and Control groups than that in the Fentanyl and Morphine groups (*P* < 0.05). In the 7–12 years old patients, the cases of used rescue medicine in the Control group were much higher than that in Fentanyl, Morphine and Tramadol groups (Additional file 1: Table S4, *P* < 0.05).

## DISSCUSION

Although research has demonstrated 41–76% of adult patients experience moderate-severe pain within 48 h after craniotomy [18, 19], very few studies have focus specifically on the incidence and treatment of POPI in pediatric neurosurgery patients [20, 21]. In this prospective, randomized, controlled clinical study conducted at a single academic hospital, we found POPI could be well controlled with opioids administration by NCIA or PCIA. Compared to the opioid groups (Fentanyl and Morphine groups), the Control group needed more rescue medicine – ibuprofen suspension or morphine. In contrast, complications during recovery such as respiration depression and consciousness changes showed no significant difference among all groups. Finally, factors such as younger age, occipital site craniotomy, Fentanyl or Morphine treatment were the relevant factors.

To date, few studies have formally recommended postoperative pain treatment protocols in pediatric neurosurgery. A prospective cohort study conducted in three academic children’s hospitals has previously reported POPI to be mild in children under various analgesic regimens [7]. However, this was a cohort study which only included where POPI was not accurately assessed. In contrast, Bronco [6]. found 16% of pediatric neurosurgical patients suffered moderate-severe pain in the recovery room, and 6% patients suffered moderate-severe pain in the first and second days after surgery despite application of multimodal analgesia [6]. The main analgesic methods advocated in current studies are multimodal analgesia and PCIA or NCIA analgesia [5, 7]. Maxwell et al. [7] have demonstrated that PCIA or NCIA analgesia is an effective analgesia with a low incidence of opioid-related side effects; although it should be noted that the analgesic pump settings in their study were not standardized. Chiaretti [22] found PCIA with fentanyl plus midazolam could effectively relieve postoperative pain in pediatric neurosurgery. However, their study only included patients over the age of 6, all of whom were managed in an ICU setting. In this study, we enrolled pediatric patients in the range of 1–12 years of age. In addition, we have implemented three different methods of analgesia to compare with control group, in order to obtain the best analgesic regimen used in children.

Our study revealed that in both younger and older pediatric patients, morphine administration was the most effective regimen of PCIA or NCIA after neurosurgery. These results are consistent with those of Warren [23] who suggested continuous morphine infusions (CMI) had an analgesic effect comparable to that of acetaminophen and codeine; yet codeine phosphate alone is typically preferred as the standard treatment for pain after cranial surgery. The Fentanyl and Tramadol groups had similar analgesic effects; echoing results by Alencar [24] in neonates. Except for nausea and vomiting, no difference was observed in the incidence of side effects, and serious side effects such as respiratory depression and altered consciousness were not observed. Respiratory depression and excessive sedation are the two most feared adverse consequences of intravenous opioid use for postoperative pain in neurosurgery; as excessive sedation affects neurological status, and respiratory depression could cause negative physiological consequences such as elevated carbon dioxide levels and alterations of cerebral perfusion and intracranial pressure. In our study, these side effects were not observed. The incidence of nausea and vomiting was not significantly higher in the morphine or fentanyl groups, but was higher in the tramadol group. In a meta-analysis of postoperative PCIA in adults, Fentanyl has been ascertained to be as effective as Tramadol, but the incidence of nausea and vomiting is higher in the Tramadol group [25]. This is similar to our results in children.

Our study is a randomized controlled trial which balanced the confounding factors well. The assessment of pain in pediatric population presents a significant challenge. Children may often be unable to accurately describe the intensity of their pain. Thus, in our study, we used 2 different pain scales suitable for each age range. In order to avoid bias, research assistants who collected postoperative pain data in our study received subspecialty training in pediatric pain assessment. All patients were followed up by the same research assistants. We found pain scores gradually decreased with time, regardless age and treatment regimen, with a small ascent occurring at 24 h. In addition, pain scores at 8, 16 and 36 h were lower than their following time point; this might be due to the fact that children were asleep at night at these points, with lower responses to pain perception. Although much rescue medicines including oral ibuprofen suspension and intravenous morphine were used in Control group compared with other groups, the POPI in Control group was still much higher than other groups, especially within the first 8 h after surgery. The morphine equivalents amount in Tramadol group was higher than that in Fentanly and Morphine groups, which may be owned to the over-estimated tramadol MME (1 mg tramadol = 0.1 mcg morphine).

We also analyzed the factors affecting postoperative pain scores by multivariate logistic regression, proving the good control of confounding factors. We found age, craniotomy site and Fentanyl and Morphine treatment were predictors of POPI. Previous studies had described POPI to vary in different craniotomy sites due to the distribution of nerve endings [5, 17, 18]. Pain scores also varied with age, this may be owned to that older children describe pain intensity with increased accuracy.

There are several limitations to our study. First, we performed local anesthesia of the surgical incision with 0.5% ropivacaine instead of scalp nerve block, a more effective auxiliary analgesia. This method may provide longer lasting analgesia in comparison to local analgesia, perhaps decreasing POPI, especially in the early postoperative period. Secondly, in our multivariate logistic regression, we found the craniotomy site was associated with postoperative pain, but a sub-group analysis was not performed as the subsamples who underwent craniotomy at different sites were relatively small. Therefore, our next step is to find more individualized analgesia regimens for patients with different craniotomy sites, in combination with scalp nerve block [26, 27].

## Conclusions

Our study indicates that factors such as younger age, occipital site craniotomy, use of fentanyl or morphine are the relevant factors for moderate-to-severe pain. PCIA or NCIA with morphine could significantly decrease postoperative pain scores without increasing the incidence of nausea, vomiting, respiratory depression and excessive sedation in pediatric patients after neurosurgery. These patients may benefit from application of our postoperative analgesia protocol.

## Additional file

Additional file 1: **Table S1.** Supplementary details for Table 2 of POPI in 1–6 years old pediatric patients. The details including 95%CI and H(K) value for pain scores among four groups by Kruskal-Wallis H-test in 1–6 years old patients. **Table S2.** Supplementary details for Table 3 of POPI in 7–12 years old pediatric patients. The details including 95%CI and H(K) value for pain scores among four groups by Kruskal-Wallis H-test in 7–12 years old patients. **Table S3.** Supplementary details for Perioperative Events Experienced For 1–6 Years Old Younger Pediatric Patients. The results of perioperative events experienced for 1–6 years old patients. The cases suffered nausea and vomiting in Tramadol group were significantly higher than that in Fentanyl, Morphine and Control groups. **Table S4.** Supplementary details for Perioperative Events Experienced For 7–12 Years Old Senior Pediatric Patients. The results of perioperative events experienced for 7–12 years old patients. There was no different in pain intensity after the removal of intubation. But the incidence of nausea in Tramadol group were much higher than that in Fentanyl, Morphine and Control groups. **Figure S1.** Comparison of post-operative pain intensity of FLACC in patients aged 1–6 years among the four study groups. The FLACC in 1–6 years old pediatric patients, over time, the pain intensity gradually decreased, and increased slightly at 24 h after surgery. **Figure S2.** Comparison of post-operative pain intensity of WBFS in patients aged 1–6 years among the four study groups. The WBFS in 1–6 years old pediatric patients, the pain score gradually decreased within 8 h after surgery, and increased slightly at 24 h. Data are presented as the mean visual analog score. **Figure S3.** Comparison of post-operative pain intensity of NRS in patients aged 7–12 years among the four study groups. The NRS in 7–12 years old pediatric patients, over time, the pain intensity gradually decreased, and increased slightly at 24 h after surgery. Data are presented as the mean visual analog score. **Figure S4.** Comparison of post-operative pain intensity of WBFS in in patients aged 7–12 years among the four study groups. The WBFS in 7–12 years old pediatric patients, the pain score gradually decreased within 8 h after surgery, and increased slightly at 24 h. Data are presented as the mean visual analog score. (DOCX 15891 kb)

## Abbreviations

ARP: Invasive arterial pressure
BP: Non-invasive blood pressure
FLACC: Faces, Legs, Activity, Cry and Consolability Scale
HR: Heart rate
MME: Milligram morphine equivalents
NCA: Nurse-controlled analgesia
NRS: Numeric rating scale
PCA: Patient-controlled analgesia
P_ET_CO_2_: End-tidal carbon dioxide partial pressure
POPI: Postoperative acute pain intensity
SpO_2_: Pulse oximetry
WBFS: Wong-Baker Faces Scale
