# Supplementary material for: Postoperative analgesia for pediatric craniotomy patients: a randomized controlled trial
Source: BMC Anesthesiol. 2019 Apr 11;19:53. doi: 10.1186/s12871-019-0722-x (PMC6458833; doi:10.1186/s12871-019-0722-x)
Supplement: Supplementary file 1 — Table S1. Supplementary details for Table 2 of POPI in 1–6 years old pediatric patients. The details including 95%CI and H(K) value for pain scores among four groups by Kruskal-Wallis H-test in 1–6 years old patients. Table S2. Supplementary details for Table 3 of POPI in 7–12 years old pediatric patients. The details including 95%CI and H(K) value for pain scores among four groups by Kruskal-Wallis H-test in 7–12 years old patients. Table S3. Supplementary details for Perioperative Events Experienced For 1–6 Years Old Younger Pediatric Patients. The results of perioperative events experienced for 1–6 years old patients. The cases suffered nausea and vomiting in Tramadol group were significantly higher than that in Fentanyl, Morphine and Control groups. Table S4. Supplementary details for Perioperative Events Experienced For 7–12 Years Old Senior Pediatric Patients. The results of perioperative events experienced for 7–12 years old patients. There was no different in pain intensity after the removal of intubation. But the incidence of nausea in Tramadol group were much higher than that in Fentanyl, Morphine and Control groups. Figure S1. Comparison of post-operative pain intensity of FLACC in patients aged 1–6 years among the four study groups. The FLACC in 1–6 years old pediatric patients, over time, the pain intensity gradually decreased, and increased slightly at 24 h after surgery. Figure S2. Comparison of post-operative pain intensity of WBFS in patients aged 1–6 years among the four study groups. The WBFS in 1–6 years old pediatric patients, the pain score gradually decreased within 8 h after surgery, and increased slightly at 24 h. Data are presented as the mean visual analog score. Figure S3. Comparison of post-operative pain intensity of NRS in patients aged 7–12 years among the four study groups. The NRS in 7–12 years old pediatric patients, over time, the pain intensity gradually decreased, and increased slightly at 24 h after surgery. Data are presented [file 12871_2019_722_MOESM1_ESM.docx]

***List of Additional Digital Content:***

1. ***Additional Table 1***. Supplementary details for table 2 of POPI in 1-6 years old pediatric patients.
2. ***Additional Table 2***. Supplementary details for table 3 of POPI in 7-12 years old pediatric patients.
3. ***Additional Table 3.*** Supplementary details for Perioperative Events Experienced For 1-6 Years Old Younger Pediatric Patients
4. ***Additional Table 4***. Supplementary details for Perioperative Events Experienced For 7-12 Years Old Senior Pediatric Patients
5. ***Additional Figure 1.*** Comparison of post-operative pain intensity of FLACC in patients aged 1-6 years among the four study groups.
6. ***Additional Figure 2****.* Comparison of post-operative pain intensity of WBFS in patients aged 1-6 years among the four study groups.
7. ***Additional Figure 3****.* Comparison of post-operative pain intensity of NRS in patients aged 7-12 years among the four study groups.
8. ***Additional Figure 4****.* Comparison of post-operative pain intensity of WBFS in in patients aged 7-12 years among the four study groups.

***Additional Table 1.*** Supplementary details for table 2 of POPI in 1-6 years old pediatric patients.

| **POPI** | **WBFS** | | **FLACC** | |
| --- | --- | --- | --- | --- |
|  | H（K） | 95%CI 1 | H（K） | 95%CI |
| **1h** | 16.153 | 0.000-0.019 | 17.291 | 0.000-0.019 |
| **2h** | 17.797 | 0.000-0.019 | 23.922 | 0.000-0.019 |
| **4h** | 13.821 | 0.000-0.018 | 17.969 | 0.000-0.019 |
| **8h** | 14.148 | 0.000-0.018 | 10.437 | 0.001-0.049 |
| **16h** | 7.390 | 0.030-0.108 | 7.887 | 0.012-0.075 |
| **24h** | 7.976 | 0.030-0.108 | 12.516 | 0.000-0.018 |
| **36h** | 12.965 | 0.000-0.019 | 6.664 | 0.054-0.146 |
| **48h** | 7.907 | 0.008-0.067 | 8.655 | 0.012-0.075 |

***CI 1*:** 95% Confidence interval for WBFS score among four groups by Kruskal-Wallis H-test;

***CI 2*:** 95% Confidence interval for NRS score among four groups by Kruskal-Wallis H-test.

***Additional Table 2.*** Supplementary details for table 3 of POPI in 7-12 years old pediatric patients.

| **POPI** | **WBFS** | | **NRS** | |
| --- | --- | --- | --- | --- |
|  | H（K） | 95%CI | H（K） | 95%CI |
| **1h** | 13.030 | 0.000-0.021 | 10.815 | 0.000-0.044 |
| **2h** | 6.713 | 0.038-0.129 | 6.598 | 0.018-0.094 |
| **4h** | 20.490 | 0.000-0.021 | 18.422 | 0.000-0.021 |
| **8h** | 13.414 | 0.000-0.021 | 16.841 | 0.000-0.021 |
| **16h** | 8.736 | 0.001-0.055 | 10.312 | 0.000-0.021 |
| **24h** | 2.128 | 0.500-0.661 | 1.609 | 0.507-0.668 |
| **36h** | 1.951 | 0.485-0.648 | 2.701 | 0.346-0.508 |
| **48h** | 6.611 | 0.044-0.138 | 7.454 | 0.023-0.103 |

***CI 1*:** 95% Confidence interval for WBFS score among four groups by Kruskal-Wallis H-test;

***CI 2*:** 95% Confidence interval for NRS score among four groups by Kruskal-Wallis H-test.

***Additional Table 3.*** Supplementary details for Perioperative Events Experienced For 1-6 Years Old Younger Pediatric Patients

|  | **Group C** | **Group F** | **Group M** | **Group T** | **X2** | **95%CI** |
| --- | --- | --- | --- | --- | --- | --- |
| **Anesthesia recovery events in PACU #** |  |  |  |  |  |  |
| Nausea(n/%) | 6/15% | 8/20% | 12/30% | 5/12.5% | 4.601 | 0.155-0.283 |
| Vomiting(n/%) | 3/7.5% | 3/7.5% | 2/5% | 0/0 | 3.158 | 0.435-0.590 |
| **Pain score after removal of tracheal intubation ∆** (WBFS, median (IQR)) | 1 (0, 2) | 1 (0, 2) | 1 (0, 2) | 1 (0, 2) | 0.344  （H（K）） | 0.960-1.000 |
| **Perioperative Events within 48h#** |  |  |  |  |  |  |
| Nausea(n/%) | 4/10% | 3/7.5% | 3/7.5% | 11/27.5% ***** | 9.812 | 0.000-0.030 |
| Vomiting(n/%) | 8/20% | 2/5% | 4/10% | 19/47.5% ***** | 26.380 | 0.000-0.019 |
| Consciousness change(n/%) | 1 / 2.5% | 5/12.5% | 7/17.5% | 9/22.5% | 7.378 | 0.021-0.092 |
| **Number of PCA presses #** (valid/invalid) | 9/5 | 9/19 | 5/0 | 6/5 | 9.641 | 0.000-0.050 |

* *P*<0.05, # Pearson’s chi-squared test of proportion, ∆ Kruskal-Wallis H-test.

PACU=post-anesthesia care uint, IOR= interquartile range

The incidence of nausea and vomiting was higher in the tramadol group than Group C (*P* <0.05);

***Additional Table 4.*** Supplementary details for Perioperative Events Experienced For 7-12 Years Old Senior Pediatric Patients

|  | **Group C** | **Group F** | **Group M** | **Group T** | **X2** | **95%CI** |
| --- | --- | --- | --- | --- | --- | --- |
| **Anesthesia recovery events in PACU #** |  |  |  |  |  |  |
| Nausea(n/%) | 8/20% | 6/15% | 3/7.5% | 3/7.5% | 4.114 | 0.217-0.358 |
| Vomiting(n/%) | 3/7.5% | 1 / 2.5% | 1 / 2.5% | 2/5% | 1.643 | 0.787-0.900 |
| **Pain score after removal of tracheal intubation ∆** (WBFS, median (IQR)) | 1 (0, 2) | 2 (1, 2) | 0 (0, 2) | 2 (0, 2) | 0.925（H（K）） | 0.787-0.905 |
| **Perioperative Events within 48h#** |  |  |  |  |  |  |
| Nausea(n/%) | 1 / 2.5% | 10/25% | 4/10% | 17/42.5% ***** | 23.438 | 0.000-0.019 |
| Vomiting(n/%) | 25/62.5% | 8/20% | 9/22.5% | 26/65% ***** | 3.463 | 0.473-0.627 |
| Consciousness change(n/%) | 1/2.5% | 3/7.5% | 0/40 | 2/5% | 29.668 | 0.000-0.019 |
| **Number of PCA presses** **#** (valid/invalid) | 6/0 | 4/0 | 3/0 | 3/3 | 7.719 | 0.000-0.153 |

* *P*<0.05, # Pearson’s chi-squared test of proportion, ∆ Kruskal-Wallis H-test.

PACU=post-anesthesia care uint, IOR= interquartile range

The incidence of nausea and vomiting was higher in the tramadol group than Group C (*P* <0.05);

**Additional Figures**

In order to observe the trend of the postoperative pain intensity in pediatric patients after 48h, we made figures of POPI and data was presented as the mean visual analog score.


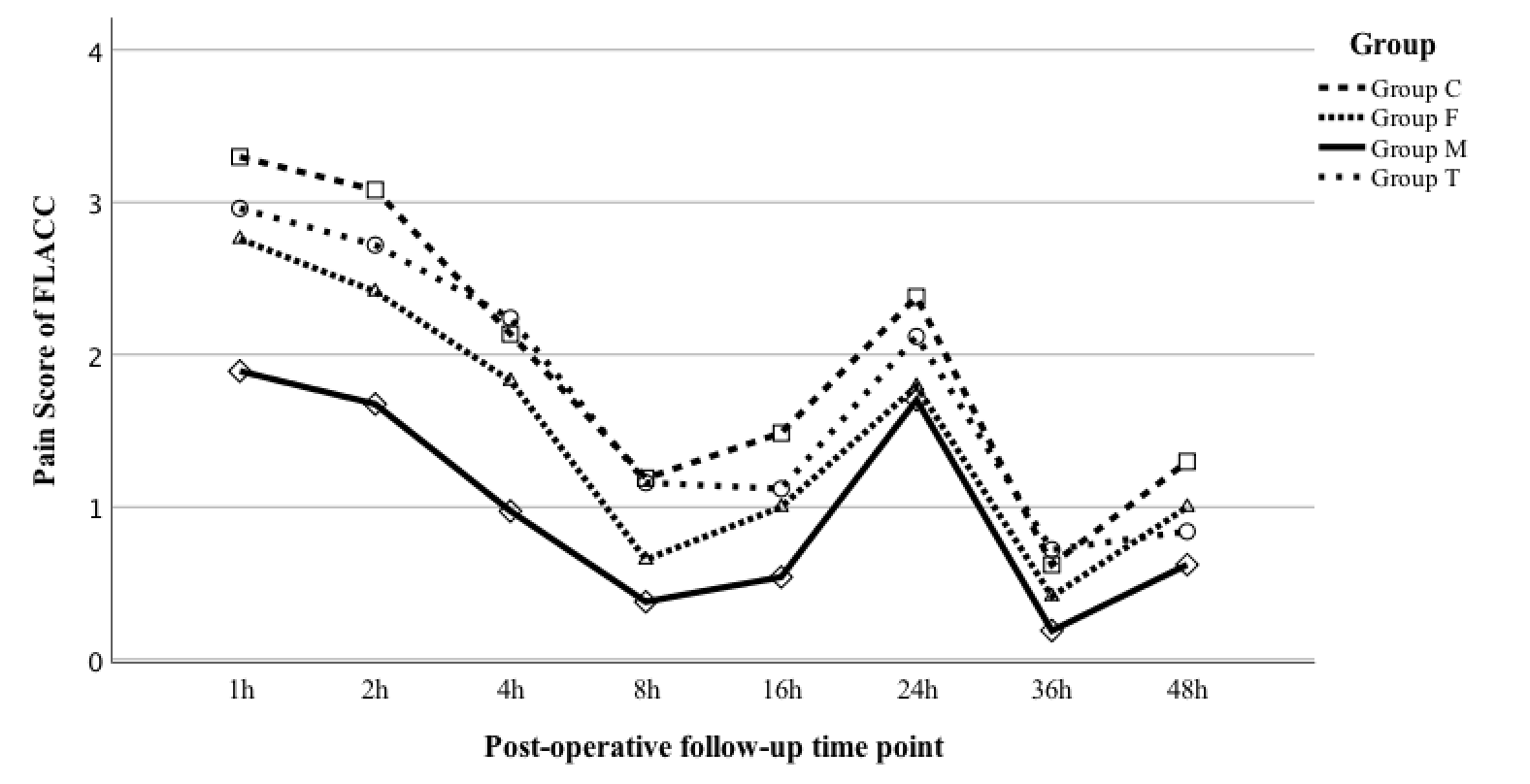


***Additional Figure 1.*** Comparison of post-operative pain intensity of FLACC in patients aged 1-6 years among the four study groups. Data are presented as the mean visual analog score.


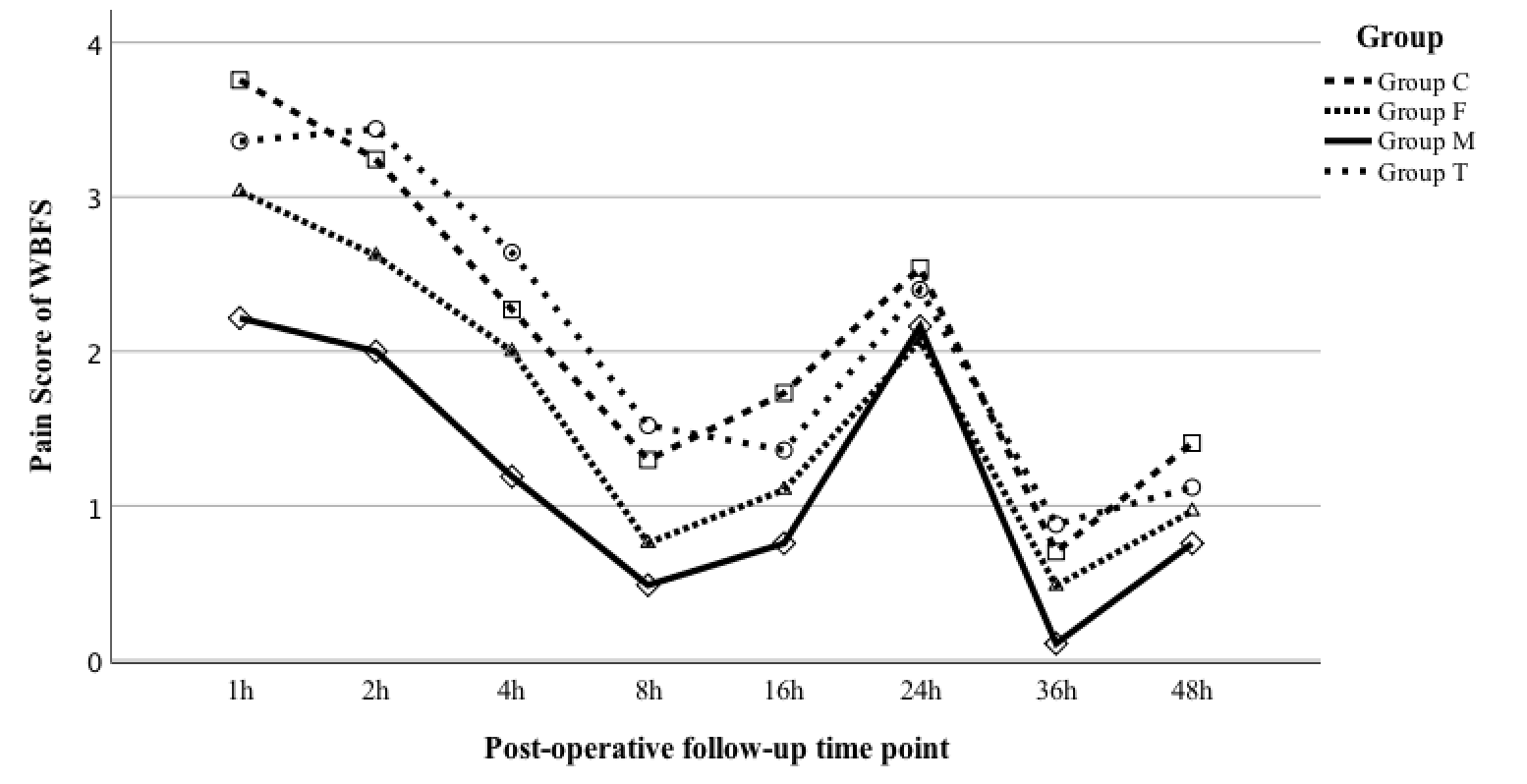


***Additional Figure 2.*** Comparison of post-operative pain intensity of WBFS in patients aged 1-6 years among the four study groups. Data are presented as the mean visual analog score.


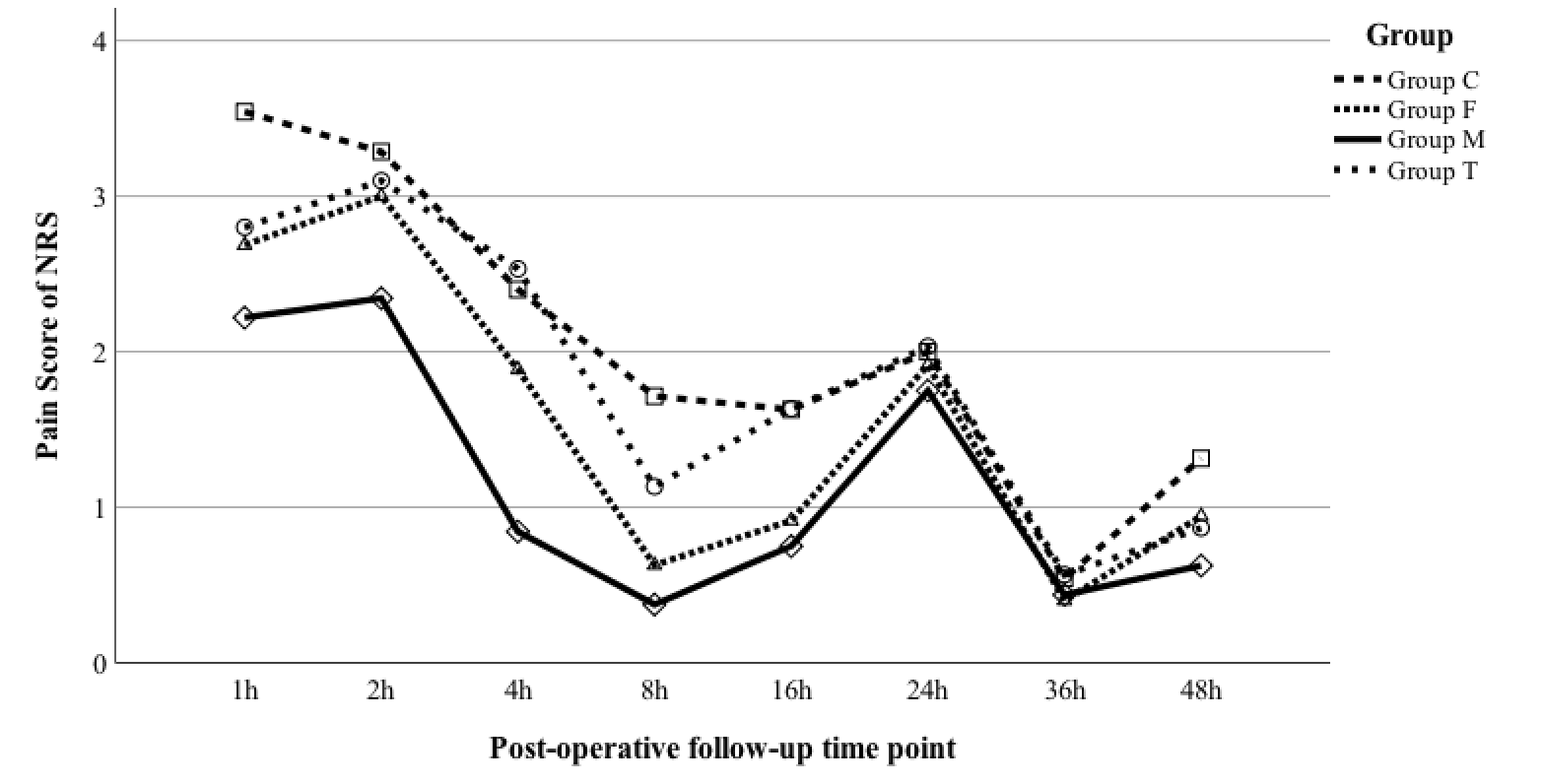


***Additional Figure 3.*** Comparison of post-operative pain intensity of NRS in patients aged 7-12 years among the four study groups. Data are presented as the mean visual analog score.


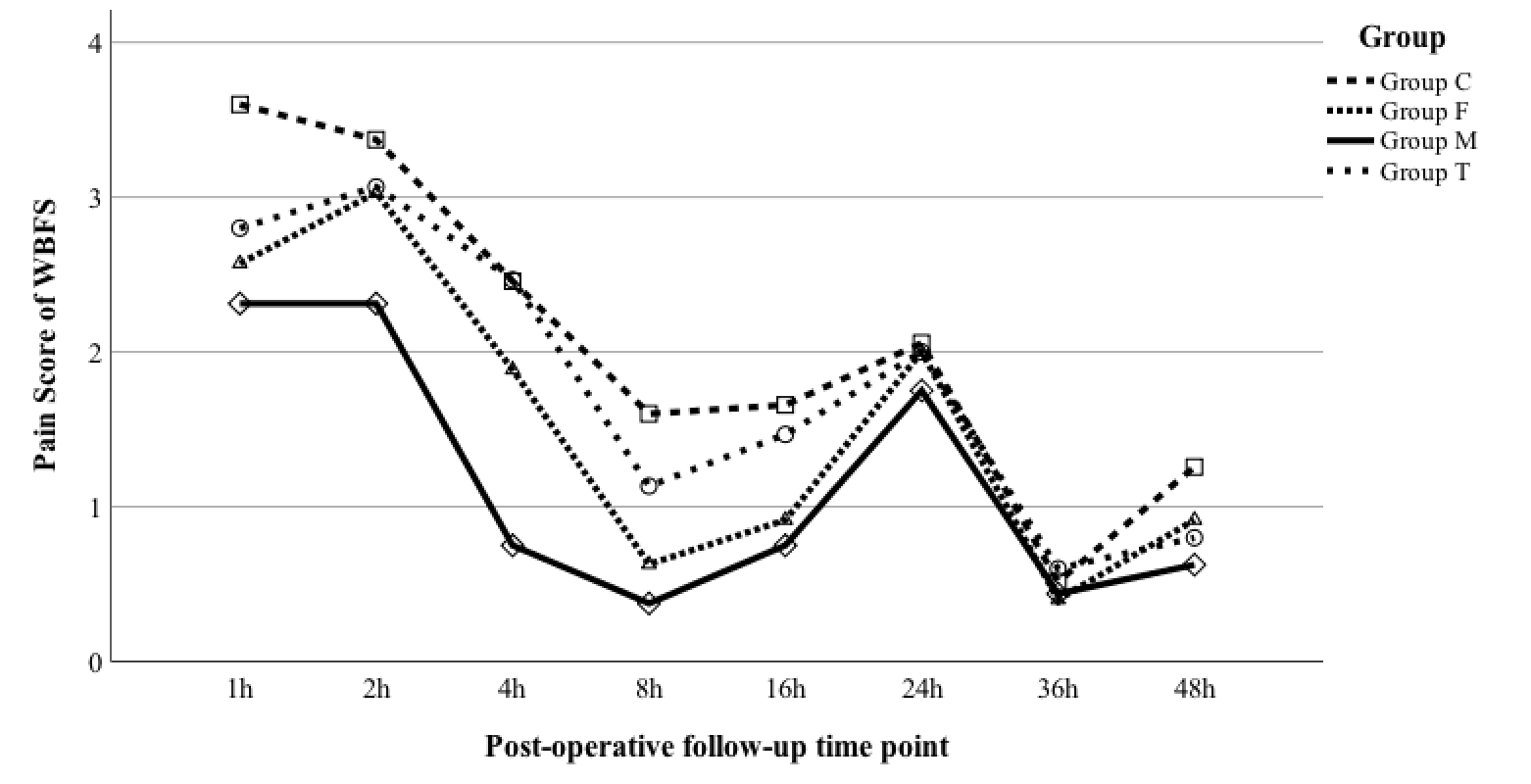


***Additional Figure 4.*** Comparison of post-operative pain intensity of WBFS in in patients aged 7-12 years. Data are presented as the mean visual analog score.
